# The Value of Ozone in CT-Guided Drainage of Multiloculated Pyogenic Liver Abscesses: A Randomized Controlled Study

**DOI:** 10.1155/2018/1236974

**Published:** 2018-03-08

**Authors:** Bing Li, Chuan Liu, Lang Wang, Yang Li, Yong Du, Chuan Zhang, Xiao-xue Xu, Han Feng Yang

**Affiliations:** Sichuan Key Laboratory of Medical Imaging, Department of Radiology, Affiliated Hospital of North Sichuan Medical College, Nanchong City, Sichuan Province 637000, China

## Abstract

**Objective:**

This study was designed to compare the effects of catheter drainage alone and combined with ozone in the management of multiloculated pyogenic liver abscess (PLA).

**Methods:**

The prospective study included 60 patients diagnosed with multiloculated PLA. All patients were randomly divided into two groups: catheter drainage alone (group I) and catheter drainage combined with ozone (group II). Drainage was considered successful when (1) the abscess cavity was drained and (2) clinical symptoms were resolved. Kruskal-Wallis nonparametric test was used to compare the success rates, length of stay (LOS), and need for further surgery of the two groups. *P* < 0.05 indicates significant difference.

**Results:**

All patients' catheters were successfully placed under CT guidance. Group I was treated with catheters alone and group II was treated with catheters and ozone. The success rates of groups I and II were 86% and 96%, respectively (*P* < 0.05). And compared with group II, the duration of fever in group I was longer (*P* < 0.05), and the LOS was also longer (*P* < 0.05).

**Conclusion:**

Catheter drainage combined with ozone is an effective and safe treatment in multiloculated PLA. The Clinical Registration Number is ChiCTR1800014865.

## 1. Introduction

Therapy of pyogenic liver abscess (PLA) includes antibiotics alone or in combination with percutaneous or surgical drainage [1, 2]. A number of studies have shown that percutaneous abscess drainage is effective and safe, and it is minimally invasive and does not require general anesthesia [3]. However, the optimal treatment of multiloculated abscesses is still a subject of debate, as the multiloculated PLA often contain viscid pus, or small locules of the abscesses cannot communicate with each other, which may make percutaneous drainage difficult [3–6].

Multiloculated liver abscess was defined as an abscess with enhancing internal septations [7]. Studies have been showed that the presence of multiloculated abscess lesions has been considered as one of the factors that increase the risk of percutaneous catheter drainage failure [7, 8]. And these literatures showed that the mortality rates for multiloculated pyogenic liver abscesses range from 44% to 22.1%, which was higher than single PLA [8, 9]. In addition, multiloculated PLA may need multiple percutaneous drains or surgical intervention in an attempt to achieve source control and hence some authors advocate surgical intervention [10]. Moreover, some patients with multiloculated PLA have various infections, where using antibiotics alone often has a poor effect [11]. Long-term use of antibiotics, in addition, can cause bacteria resistance [6].

Ozone can inactivate bacteria, viruses, yeasts, protozoa, and fungi and stimulate the immune system and oxygen metabolism, so ozone has been widely considered to be one of the best sterilization, antifungal, and antiviral agents [12]. For some chronic wounds, such as nutrition ulcers, ischemic ulcers, and diabetic wounds, ozone has also been empirically used as clinical therapeutic agent [13–15].

In order to improve the therapeutic effect of multiloculated PLA, this study intends to compare catheter drainage alone and its combination with ozone therapy in the treatment of multiloculated PLA.

## 2. Materials and Methods

This prospective study was approved by our Hospital Institutional Review Board (HIRB), which included 60 patients who were diagnosed with multiloculated PLA (32 males and 28 females, aged 37–71 years; median age of 47.4 years) between January 2014 and October 2017. All patients had given written consent for this study. Diagnosis of the pyogenic abscess was proved by aspiration or microbiologic findings in all patients. Patients with cancer or diabetes were excluded from this study.

Inclusion criteria were as follows:

(1) Multiloculated PLA more than 5 cm in size

(2) Multiloculated PLA in mature stage (a capsule was formed around the necrotic cavity)

Exclusion criteria were as follows:

(1) Multiple abscesses

(2) Abscess ruptured into thoracic and peritoneal cavity

All patients have been uniformly managed with antibiotics throughout the study period, usually a combination of a third-generation cephalosporin along with metronidazole. Subsequently antibiotic therapy is tailored according to culture and sensitivity results of blood or pus.

Patients were randomly allocated into two groups with the help of a computer-generated table of random numbers: catheter drainage alone (group I) and catheter drainage combined with ozone (group II). Contrast-enhanced CT scans were performed in all patients to determine the size, location, and extent of the lesion and choose transhepatic route to avoid injuring other organs, blood vessel, and biliary systems. All catheters were placed by CT guidance, performed under local anesthesia. 10F pigtail catheters were placed into the patients by using the Seldinger technique. Attention should be taken to ensuring the side holes of catheter were placed within the abscess cavity. In this way, we avoid secondary liver infection. Abdominal CT images were obtained immediately after abscess drainage to assess the catheter location, abscess cavity, and complications such as bleeding. Then the valve of catheter was unclamped for open drainage.

In group II, in addition to drainage, according to the size of the abscess cavity, 10.0–20.0 mL oxygen-ozone gas mixture (ozone concentration 25 *μ*g/ml, based on our previous experiments [12]) was given through catheter, until the amount of drainage was less than 20 mL per day. We used an ozone generator (Herrmann, Kleinwallstadt, Germany) to produce oxygen-ozone gas mixtures prior to injection. After oxygen-ozone gas mixture injection, the catheter was clamped for one hour and then left unclamped for 23 hours to allow for open drainage.

The drainage was considered successful when clinical symptoms of patients were resolved and the abscess cavities were drained. And the patients were referred for further surgical treatment when (1) the abscess failed to resolve, (2) the follow-up imaging (ultrasound or CT) showed that the abscess wall becomes thicker and cannot be aspirated, or (3) the patients have ongoing sepsis after drainage.

We evaluated the patient characteristics differences between the two groups and the technical success of catheter placement, as well as the length of hospital stay (LOS). Clinical details such as patients with ongoing sepsis, duration of fever (morning oral temperature of >37.5°C), and patients who converted into further surgery treatment were also written down.

We used SPSS software (version 20.0; SPSS Corporation, New York, USA) for statistical analyses of the two groups. The mean and standard deviation (SD) of each variable from two groups were calculated. Kruskal-Wallis nonparametric test was used to calculate statistical differences between two groups, with a *P* value of less than 0.05 considered significant. And the descriptive statistical analysis was calculated too.

## 3. Results

**Figure 1:**
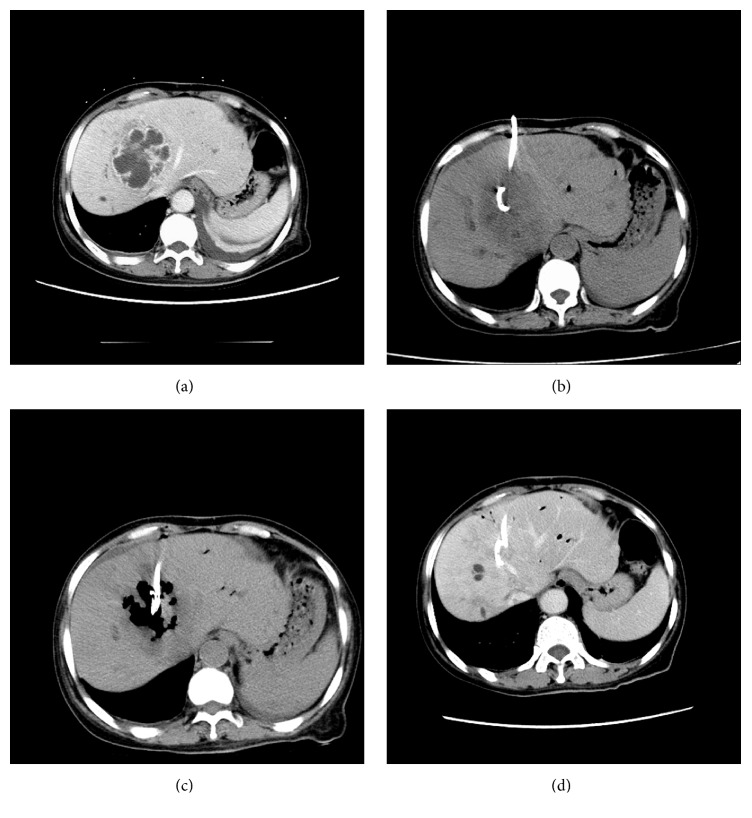
A 49-year-old male presented with fever and right back pain for one week. (a) A lager multiloculated liver abscess in the right hepatic lobes was shown by enhanced CT, and small amount of fluid can be seen in the left chest. (b) Catheter was placed under CT guidance, without injection of oxygen-ozone gas. (c) Three-week follow-up showing that abscess was not fully absorbed. (d) Abscess was fully absorbed after eight weeks.

**Figure 2:**
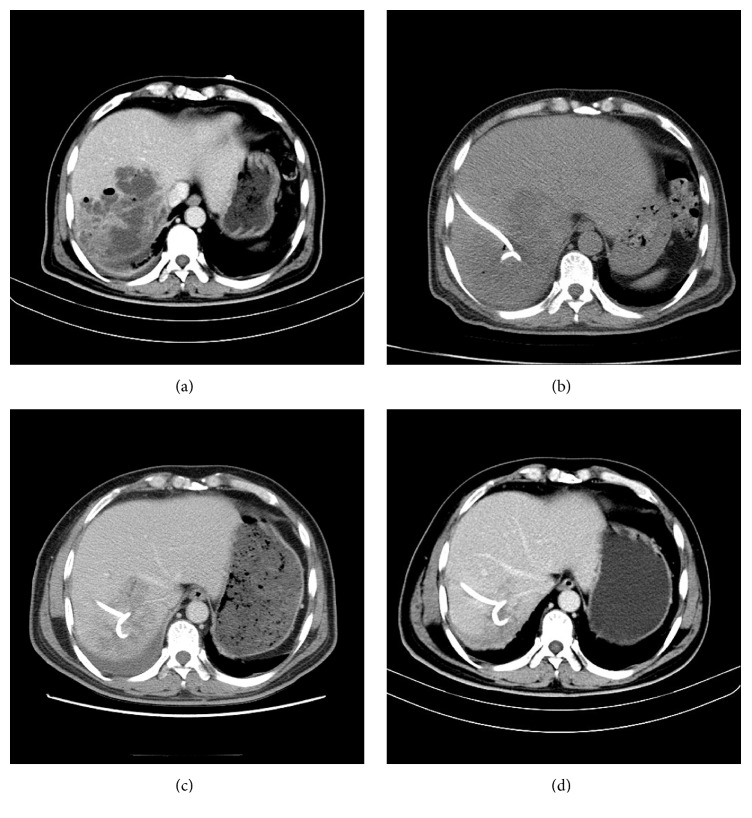
A 43-year-old male presented with fever and right abdominal pain for five days. (a) Enhanced CT shows a lager multiloculated liver abscess in the right hepatic lobe. The abscess cavity has gas formation, which is associated with mortality. (b) Catheter was placed under CT guidance. (c) After the pus was pulled out, oxygen-ozone gas mixture was given through catheter and filled the small locules of the abscesses; some separation was broken. (d) Abscess was absorbed after three weeks.

All of the 60 patients' catheters were successfully placed under CT guidance. Group I was treated with catheters alone (Figure 1) and group II was treated with catheters and ozone (Figure 2). Different variables of the two groups are all shown in Table 1. Mean age, abscess sizes, and number of two groups were not significantly different (*P* = 0.437; *P* = 0.471; Table 1). Among the 60 patients, bacteriologic study of cultures had positive findings in 100% of patients; some patients have mixed infection (Table 2). The remaining abscesses were diagnosed based on increased white blood cell count in the aspirated fluid but had negative findings on culture. Streptococcus species were the main bacteria isolated, followed by* Enterococcus* species,* Escherichia coli*, and* Klebsiella* species.

Initial white blood cell count (WBC), lymphocytes, and duration of symptoms were different in two groups but were not significant (*P* > 0.05). Success rates of groups I and II were 86% and 96%, respectively. And compared with group II, the duration of fever in group I was longer (*P* < 0.05), and the LOS was also longer (*P* < 0.05). Furthermore, there were significant differences when comparing patients who converted into further surgery between the two groups (14% versus 4%; *P* < 0.05) (Table 3). Complications included three patients with minimal perihepatic bleeding. No other complications were founded.

## 4. Discussion

Despite advances in diagnostic technology and new strategies for treatment, PLA remains a big therapeutic challenge. Conventional treatment of PLA is antibiotic therapy and image-guided percutaneous drainage or aspiration [16]. However, the study reported that percutaneous catheter drainage still has high failure rates, especially in patients with multiloculated PLA [8, 17]. The possible reason behind such high failure rate is either the presence of viscid pus within the abscesses or the inability of multiple small locals of the abscesses to fuse together and communicate with each other [2]. And no matter any intervention type for an abscess was used, the bacteria may release into the bloodstream [18]. Some studies have showed the chance of postprocedure sepsis after liver abscess drainage [7, 16, 19].

Our study used catheter drainage combined with ozone to improve the effects of treatment for multiloculated PLA and achieved relatively higher success rates (96%) than when using catheter drainage only [16, 20]. And the rates of ongoing sepsis and LOS were decreased significantly.

The operation of this study was carried out under CT guidance. CT imaging can more accurately display the lesion and the septae of the abscess, which is critical to the success rate of treatment. And we used a Seldinger technique which a guide wire was placed through abscess cavities; in this way, the septae of abscess may disrupt, and then the abscess can be drained more effectively. Through this method, compared with the standard catheter drainage treatment, we can get a better therapeutic effect. Abscesses with small collections and with presence of air as well as those closely abutting the diaphragm are poorly defined on US. CT imaging was found to be superior in depicting such abscesses [21].

Clinical application has proven that ozone is a powerful and reliable antimicrobial agent and can inactivate bacteria, fungi, protozoa, and viruses [12, 22, 23]. From a century ago, ozone therapy has been widely used and studied. Its mechanism action is through stimulating the oxygen metabolism and the immune system of bacteria, viruses, fungi, yeast, and protozoa, to achieve inactivation [12, 22, 23].

Using antibiotics only sometimes cannot achieve satisfactory results as most multiloculated PLA patients have mixed infections. In addition, long-term use of antibiotics can lead to bacterial resistance [2, 6, 24]. And the excess and indiscriminate use of antimicrobial drugs appears to be the most significant factor in the emergence of resistant microorganisms in recent years. In 2016, a global alliance consists of a multidisciplinary task force from 79 different countries developing a consensus on the rational use of antimicrobials for patients with intra-abdominal infections (IAIs). The consensus demonstrates the necessity of a multidisciplinary and collaborative approach in the battle against antimicrobial resistance in surgical infections. The use of ozone as a secondary antibacterial agent to treat multiloculated PLA has achieved a better effect.

Identifying pathogenic organisms has a great clinical importance. Experiences, however, have shown that this was not always possible because many patients were treated before fluid was obtained for culture [2]. Therefore, the blood culture of some patients in two groups of our study was negative. We observe that commonest organism in our study is neither* Klebsiella* nor* E. coli *and this is contradictory to global reports [25]. Prophylactic antibiotics for* E. coli* are usually used in clinical practice in our institution. This may result in negative bacteria culture in some patients who were originally infected with* E. coli*. There was a decreased mortality in culture negative PLA patients who receive percutaneous drainage. And there was no difference in outcomes of percutaneous drainage between* E. coli* PLA and* Klebsiella* PLA.

The study found that the beneficial effects of ozone on the wound healing might be assumed due to increased oxygen tension by ozone exposure in the wound area and then ameliorated impaired wounds healing [23]. Another study reported that ozone exposure could activate transcription factors. And this is important to adjust inflammatory reactions and eventually the whole process of wounds healing [22]. Therefore, ozone has also been used empirically as a clinical therapeutic agent for chronic wounds, such as ischemic ulcers, trophic ulcers, and diabetic wounds [26]. Furthermore, ozone can be dispersed into the cavity of the abscess, causing abscess wall dehydration [15]. Moreover, injection of ozone can break the septae of abscess and separate the adhesion in abscess cavity to make the drainage more effective (Figure 2) [12]. So the use of drainage combined with ozone has a synergistic effect on the management of liver abscess.

The categories of PLA often included biliary infection, portal vein seeding, direct extension, hepatic arterial seeding, penetrating trauma, and cryptogenic cause [25]. In our study, the biliary obstruction with cholangitis is the most common cause of PLA. Some complications from catheter drainage, such as hemorrhage owing to intercostal vessel and liver parenchyma injury, catheter-related pain, and subcutaneous emphysema, have been reported [11]. In this study, we did not encounter any serious complications. And there was no significant clinical bleeding noted in any patient in groups I and II.

## 5. Conclusions

In short, combined treatment of catheter drainage and ozone is a safe and valid therapeutic procedure in multiloculated PLA. This technology can decrease the ratio of surgical interventions and the LOS and should be confirmed with a larger patient series in other medical institutions.

## Figures and Tables

**Table 1:** Comparison of preadmission variables among treatment groups.

Variable, mean (SD)	Group I	Group II
Number of patients	30	30
Average age (yr)	41.4 (10.4)	38.2 (13.6)
Duration of symptoms (d)	14.5 (8.9)	14.3 (7.9)
Initial WBC (×103)	14.9 (9.4)	15.4 (9.2)
Total lymphocytes (×103)	11.4 (6.7)	10.9 (6.9)
Abscess size (cm)	6.8 (2.6)	6.7 (2.5)
Catheter size (Fr)	10	10

**Table 2:** Positive results of bacterial culture among treatment groups.

Variable, mean	Group I	Group II
Bacterial culture	17	16
*Streptococcus* species	7	6
*Enterococcus* species	5	6
*Escherichia coli*	4	4
*Klebsiella* species	3	2

**Table 3:** Comparison of hospitalization and outcome variables among treatment groups.

Variable, mean (SD)	Group I	Group II
Technical success of catheter placement	100%	100%
Success rate of management	86%	96%
LOS (d)	26.1 (10.3)	21.4 (8.2)
Duration of fever (d)	5.5 (3.0)	3.5 (2.1)
Converted into further surgery	4 (14%)	1 (4%)
With ongoing sepsis	6 (20%)	1 (4%)

## Abbreviations

PLA: Pyogenic liver abscess
LOS: Length of stay
PCD: Percutaneous drainage catheter
WBC: White blood cell count.
